# Using a Descriptive Social Norm to Increase Vegetable Selection in Workplace Restaurant Settings

**DOI:** 10.1037/hea0000478

**Published:** 2017-05-25

**Authors:** Jason M. Thomas, Amanda Ursell, Eric L. Robinson, Paul Aveyard, Susan A. Jebb, C. Peter Herman, Suzanne Higgs

**Affiliations:** 1 School of Psychology, University of Birmingham, and Department of Psychology, Aston University; 2 Department of Sport and Health Sciences, Oxford Brookes University; 3 Department of Psychological Sciences, University of Liverpool; 4 Nuffield Department of Primary Care Health Sciences, Radcliffe Observatory Quarter, University of Oxford; 5 Department of Psychology, University of Toronto; 6 School of Psychology, University of Birmingham

**Keywords:** social norms, descriptive norm, healthy eating, vegetables, field study

## Abstract

***Objective:*** Recent work has shown that exposure to social norm messages may enhance the consumption of vegetables. However, the majority of this work has been conducted in laboratories, often with student populations. Little is known about whether this approach can be successfully used in other contexts. In this study, a poster featuring a message based on social norms was tested to examine whether it could increase and maintain the purchase of meals with vegetables in workplace restaurants. ***Method:*** A pretest–posttest design with 3 phases was used in 3 workplace restaurants in the United Kingdom. The first 2 weeks formed the preintervention phase, the second 2 weeks the intervention phase, and the last 2 weeks the postintervention phase. During the intervention phase only, posters containing a social norm message relaying information about vegetable purchases of other diners were placed in each restaurant. The main outcome measure was the percentage of meals purchased with vegetables, which was analyzed using Pearson’s chi-squared test. ***Results:*** Participants were judged to be male (57%), not overweight (75%), and under the age of 60 (98%). The intervention was positively associated with the percentage of meals purchased with vegetables: baseline versus intervention (60% vs. 64% of meals purchased with vegetables; *p* < .01); intervention versus postintervention (64% vs. 67% of meals purchased with vegetables; *p* < .01); and baseline versus postintervention (60% vs. 67% of meals purchased with vegetables; *p* < .001). ***Conclusions:*** Social norm messages may increase the purchase of vegetables in workplace settings.

Individuals who report higher intakes of vegetables have a lower risk of a range of conditions such as coronary heart disease, stroke, and cancer (He, Nowson, & MacGregor, 2006; Oyebode, Gordon-Dseagu, Walker, & Mindell, 2014), and population dietary guidelines recommend an increase in consumption. In the United Kingdom (U.K.), the “5 A Day” campaign has high levels of public recognition, but the intake of fruits and vegetables remains below the recommended amount (Public Health England & Food Standards Agency, 2014). A review of international campaigns to increase consumption suggests that informational campaigns have had limited success (Rekhy & McConchie, 2014).

Providing descriptive social norm information about the healthy behaviors of others has been shown to be effective in promoting health behaviors such as stair climbing and in reducing unhealthy behaviors such as binge drinking, drunk driving, smoking, and unsafe sex (Burger & Shelton, 2011; Chernoff & Davison 2005; DeJong et al., 2009; Linkenbach & Perkins, 2003; Perkins, Linkenbach, Lewis, & Neighbors, 2010). For example, some research suggests that providing students with the information that other students drink less frequently than they might think reduces levels of drinking on university campuses (for a review see Perkins, 2002). However, to date, few studies have investigated the potential of social norm messages to promote healthy eating in field settings.

There is extensive evidence from laboratory-based studies of eating behavior and from food diary studies with free-living participants, that the amount of food that people consume at an eating occasion is influenced by the eating behavior of other people (De Castro, Brewer, Elmore, & Orozco, 1990; for reviews see Herman, Roth, & Polivy, 2003; Robinson, Blissett, & Higgs, 2013; Robinson, Thomas, Aveyard, & Higgs, 2014; Vartanian, Spanos, Herman, & Polivy, 2015). For example, people tend to eat more when they dine with familiar others (Herman, 2015). There is also evidence that our perceptions of what other people eat predicts our own self-reported eating (de la Haye, Robins, Mohr, & Wilson, 2013), perhaps explaining why dietary patterns of socially connected individuals tend to be similar (Pachucki, Jacques, & Christakis, 2011). These data suggest that people use the eating behavior of others as a guide or norm and follow their lead when it comes to dietary decisions (Cruwys, Bevelander, & Hermans, 2015).

Indeed, providing information about the fruit and vegetable choices of others has been shown in experimental studies to affect eating behavior. For instance, providing social normative information about fruit and vegetables has been shown to enhance the intention to eat these foods by men (Croker, Whitaker, Cooke, & Wardle, 2009). Presenting high school students with a descriptive social norm, suggesting that a majority of high school students try to eat a sufficient amount of fruit, produced a trend for enhanced self-reported consumption of fruit over a 2-day follow-up period (Stok, de Ridder, de Vet, & de Wit, 2014). Similarly, social norms significantly enhance intentions to consume vegetables and also show a trend towards increasing self-reported vegetable intake (Stok, Verkooijen, de Ridder, de Wit, & de Vet, 2014). However, a limitation of these studies is that they focus on self-reported eating behavior and intentions to eat healthily, rather than measuring the consumption of these foods directly.

Exploring the use of social norms on actual fruit and vegetable intake in the laboratory, Robinson and colleagues conducted two studies (Robinson, Fleming, & Higgs, 2014). In the first study, exposure to a descriptive norm message suggesting that most students ate more than 3 servings of vegetables a day significantly enhanced consumption of vegetables by participants by almost half a portion at a subsequent food buffet. The second study used a descriptive norm message suggesting that most students eat their five servings of fruit and vegetables a day, which also significantly enhanced the consumption of these foods by more than half a portion when later provided at a food buffet. Notably, in both experiments by Robinson and colleagues (2014), when split by habitual consumption, low but not high consumers of fruit and vegetables showed increased consumption of these foods after they were presented with social norm information. It appears that low-consuming individuals increase consumption to become more in line with the norm, while higher consumers do not change their consumption. This suggests that the overall effect of social norm messages to enhance fruit and vegetable intake is due to effects on low consumers.

Prior to conducting the present study, to the best of our knowledge only one published study has tested the effect of social norm messages on healthy eating in restaurant settings. Mollen, Rimal, Ruiter, and Kok (2013) found that a poster displaying a healthy eating norm message increased self-reported consumption of salad in a university canteen, but this effect was observed only for those participants who recalled seeing the poster. As the work was conducted with students in a specific site (university canteen), it is unclear how effective these messages are in other settings and with other populations. Also, there are some data to suggest that behavior change in response to a norm intervention might be sustained beyond the intervention period, but this possibility has yet to be assessed for an eating behavior intervention (Lewis & Neighbors, 2007; Neighbors, Larimer, & Lewis, 2004).

In the present study, a social norm intervention was tested to examine whether it could enhance and then maintain the purchase of meals with vegetables in workplace restaurants, by adopting a method similar to that used by Burger and Shelton (2011): examining purchases during preintervention, intervention, and postintervention observation periods. It was hypothesized that introducing an accurate social norm message indicating that most diners in the restaurant consume vegetables with their lunch would be associated with an increase in the purchase of meals with vegetables. Based on the results of Burger and Shelton (2011), who found that the effects of a descriptive norm message on stair climbing was maintained a week after the poster were taken down from the site, we hypothesized that this effect might be maintained for at least a week after the posters were removed.

## Method

### Participants

Participants comprised all individuals who purchased a meal within one of the three restaurants during the study period. Ethics approval was obtained from the University of Birmingham Science, Technology, Engineering and Mathematics Review Committee (Approval code: ERN_13-0475AP8). The study was conducted in accordance with the British Psychological Society Guidelines on observational research, and informed consent was not obtained.

### Design

A pretest–posttest quasi-experimental design was used with three consecutive phases, each lasting 2 weeks: Preintervention Phase, Intervention Phase, and Postintervention Phase. During each phase cash register purchases made by participants dining at each of the three restaurants were recorded. During the intervention phase only, posters containing the social norm message were displayed in all restaurants. General posters on healthy eating that were normally displayed in the restaurants were present in all sites throughout the study.

### Sample Size

Previous work using a social norm intervention in a pretest–posttest observational design yielded small effect sizes with phi values of 0.1 (Burger & Shelton, 2011). A power analysis was conducted (G-Power 3.1) revealing that at least 785 observations were required to detect a small effect (assuming an alpha of 0.05 and power of 80).

### Social Norm Message

Posters were used to communicate the same descriptive social norm message in each restaurant. On average, five posters (210 mm × 297 mm) were placed in each restaurant, near to the entrances and on top of the food counters at the point of selection. In addition, approximately 10 smaller (148 mm × 210 mm) posters were placed at each site in table-top holders, such that approximately half of the tables in each site featured one. The posters were printed in color, featuring a brown wood effect background with text superimposed, varying in color (gray, beige, orange, blue, and white) and font type and size. Small leaf/floral motifs (white) were incorporated into the top and bottom of the poster design (above and below the text). The message stated, “Most people here choose to eat vegetables with their lunch.”

The message was based on data acquired over a 2-week period prior to the start of the study at each site, using the same approach used for the study phases. The majority of participants (62%) purchased meals containing vegetables across all three sites. The poster message and design of the poster were selected on the basis of the responses of a focus group with 12 participants.

### Restaurants and Meals

Three restaurants were recruited to this study. Two were based in the South of England and one in South Wales, U.K. Data collection was carried out between February and August, 2015; none of the 6-week data collection periods included any public holidays. All three sites were workplace restaurants, with two serving meals seven days a week (from early morning to late night), and the other restaurant serving meals Monday to Friday only (from morning to midafternoon). All three catering sites were run by the same external catering company. Each site was a self-service restaurant serving hot and cold food and drink. Diners queued to select food at a food counter and then purchased their selection at the cash register before sitting to eat. All sites offered a variety of main meals that included meals served with and without vegetables. These included hot meals (e.g., fish and fries) and cold meals (e.g., salads). Most meals cost between £3 and £4 (Great Britain Pound—GBP). All sites also offered side portions of vegetables, and it was possible for people to add vegetables to a meal that might otherwise not include them. They could also purchase other food items (e.g., cakes and chips) and drinks (e.g., water, soft drinks, and hot drinks). Communal tables were provided for dining. The tables seated groups of around 2–6 people.

### Procedure

#### Cash registers

All meal purchases were automatically recorded by cash registers at each of the restaurants, providing purchase data for all meals over the entire day. At one restaurant, cash register operators recorded lunchtime meal purchases by pressing the appropriate cash register button to indicate the specific meal that was purchased. Side orders of vegetables were also recorded by pressing a separate button. The chef at the restaurant was able to indicate to researchers which meals contained at least one serving of vegetables (at least 80g), thereby enabling the identification of these meals. At the two remaining restaurants, chefs directly indicated to the operators which meals contained at least 80g of vegetables. During each purchase, operators were then able to directly indicate whether lunchtime meals contained or did not contain an 80g portion of vegetables, by pressing the corresponding button on the cash register operating system. They were also able to indicate side orders of vegetables by pressing a separate button. Researchers also trained the operators at these two restaurants to ensure consistent and correct identification of meals with and without vegetables. For the purposes of this study, vegetables included leaf (e.g., spinach), pod (e.g., peas), legumes (e.g., lentils), root (e.g., carrots), bulb/stem (e.g., onion), and flower vegetables (e.g., broccoli). Potatoes (in any configuration) and vegetable-based garnishes (e.g., a single leaf of lettuce) were not counted. Sales at each site were monitored against stock, and none of the sites reported any significant discrepancies between these figures, which suggests that the till receipts were an accurate reflection of actual sales. The data from the tills were comprehensive in providing detailed information about all sales.

#### Observation of participant characteristics

For one day during each phase at each restaurant a pair of researchers observed participants purchasing meals in the restaurants to estimate basic demographics (gender, age, and weight status). Researchers were blinded to each other’s ratings. Observations were conducted from 11:00 a.m. to 2:00 p.m. Using previously established criteria (Eves, Webb, & Mutrie, 2006; Kerr, Eves, & Carroll, 2001), participant gender was categorized on visual appraisal, weight status was categorized using body silhouettes (nonoverweight vs. those who were overweight or greater), and age (under 60 vs. over 60) was categorized based upon presence of gray hair and general appearance.

### Analysis

#### Participant characteristics

To assess interrater reliability between observers, kappa max was used, reflecting the small differences in the total number of observations made by researchers. Data on participant characteristics were combined across all sites and analyzed with Pearson’s chi-squared test in the International Business Machines Corporation Statistical Package for the Social Sciences (IBM SPSS, version 20), comparing (a) preintervention to intervention, and (b) intervention to postintervention. This provided a check of whether the people visiting the restaurants changed between the phases.

#### Meal data

The number of meals purchased with and without vegetables during each 2-week period were recorded at each site and combined across all sites. Pearson’s chi-squared test was used to compare (a) preintervention to intervention, (b) intervention to postintervention, and (c) preintervention to postintervention (to examine meal selections across the study). Odds ratio (*OR*) and confidence intervals (CI) were also estimated.

#### Cold drinks data

Purchases of water (as a percentage of the total number of cold drink purchases) were also examined as a comparator. This was to check whether purchases that were not expected to change across phases (because they were not the target of the social norm messages) changed, possibly because of a general change in purchasing patterns unrelated to the intervention. The data were extracted using the same approach used for the meal data.

## Results

### Participant Characteristics

Observers showed very good interobserver reliability (Landis & Koch, 1977), with a mean kappa max coefficient of 0.92 (range = 0.87 to 0.99). In total, 1585 participant observations were dual coded. Participant characteristics were averaged across sites; overall, 57% of those observed were men, 75% were judged not to be overweight or obese, and 98% of participants were under 60 years of age. Examination of each characteristic across the different phases using Pearson’s chi-squared test revealed that observed participant characteristics did not significantly differ across the test phases (all *p*s > .05; see Table 1 for a breakdown by study phase).[Table-anchor tbl1]

### Meal Purchases

A total of 9445 meal purchases were recorded (further details are outlined in Table 1). The overall number of meals purchased remained stable over time, but the intervention and postintervention phases were associated with increased purchase of meals with vegetables.

**Figure 1 fig1:**
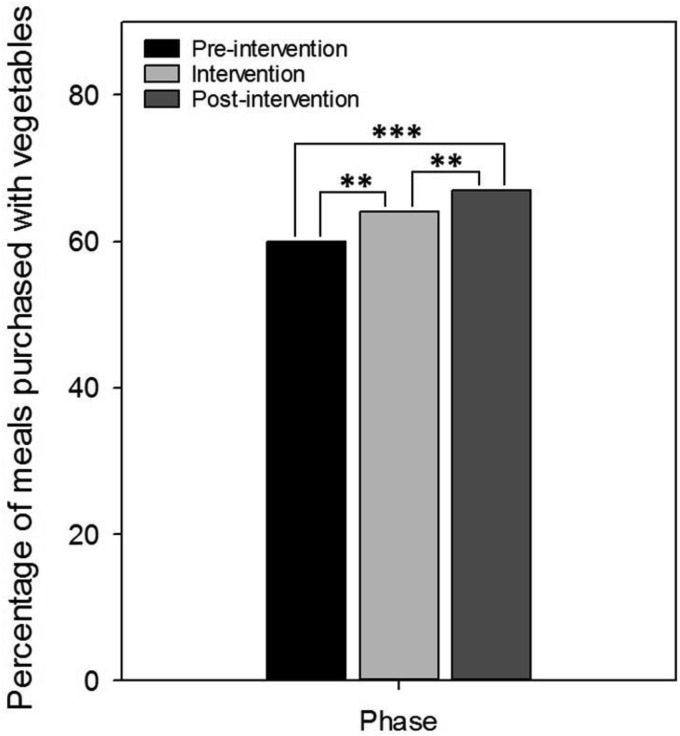
Percentage of meals purchased with vegetables, split by test phase. The introduction of the poster was associated with a significant increase of meals purchased with vegetables from the preintervention to intervention phase. Removal of the poster was associated with a further increase from the intervention to postintervention phase. Overall, baseline to postintervention was associated with an increase in the purchase of meals containing vegetables. ** *p* < .01. *** *p* < .001.

Pearson’s chi-squared test revealed that the introduction of the posters was associated with an increase in purchase of meals with vegetables from 60% during preintervention to 64% during the intervention phase; χ^2^(1, *N* = 6,357) = 11.32, *p* < .01, Φ = 0.04 (*OR* 1.2, 95% CI [1.1–1.3]). From the intervention phase to the postintervention phase there was a further increase in purchase of meals with vegetables from 64% to 67%; χ^2^(1, *N* = 6,267) = 7.27, *p* < .01, Φ = 0.03 (*OR* 1.2, 95% CI [1.0–1.3]). Overall, preintervention to postintervention was associated with an increase in the purchase of meals with vegetables from 60% to 67%; χ^2^(1, *N* = 6,266) = 36.35, *p* < .001, Φ = 0.08 (*OR* 1.4, 95% CI [1.2–1.5]) (See Figure 1).[Fig-anchor fig1]

### Cold Drink Purchases

A total of 15,415 cold drink purchases were recorded (further details outlined in Table 1). Pearson’s chi-squared test revealed that there was no significant association between test-phase and purchase of water from preintervention to the intervention phase; 15% vs. 14%; χ^2^(1, *N* = 11,669) = 3.22, *p* > .05, Φ = 0.02 (*OR* 0.9, 95% CI [0.8–1.0]); intervention phase to the postintervention phase; 14% vs. 14%; χ^2^(1, *N* = 10,520) = 0.95, *p* > .05, Φ = 0.00 (*OR* 1.0, 95% CI [0.9–1.1]); or the preintervention phase to the postintervention; 15% vs. 14%; χ^2^(1, *N* = 10,123) = 2.21, *p* > .05, Φ = 0.02 (*OR* 0.9, 95% CI [0.8–1.0]); see Table 1.

## Discussion

The introduction of posters displaying social norm messages emphasizing that most people eat vegetables with their meal in a workplace restaurant was associated with an increase in the proportion of meals purchased with vegetables, compared to the baseline period. The influence of the poster on the purchase of meals with vegetables persisted after the removal of the poster. This study suggests that social norms might be used to promote the selection of vegetables in a real-world context.

It was hypothesized that the social norm message would be associated with an increase in the purchase of meals with vegetables because diners would use the information in the message as a guide to appropriate behavior in that context (Herman et al., 2003). People tend to follow group norms because they provide a useful guide as to “correct” behavior (everyone else is behaving this way for a reason so it is probably a good idea for me to behave similarly; see Robinson et al., 2013, for a review), but following a norm is also a positive experience because it enhances affiliation with the group and/or avoids negative sanctions associated with not conforming to the group norm (Higgs, 2015). The mechanism underlying behavior change is unclear but one possibility is that the provision of the normative information led customers to compare themselves to the norm, which for some customers highlighted their deviation from the norm, leading to a change in behavior to bring them more in line with the perceived norm (Higgs & Thomas, 2016; Polivy & Pliner, 2015). The poster may have brought into focus the normative information and/or corrected a misperception about the norm. In support of this idea, correcting misperceptions of excess alcohol consumption has been shown to reduce subsequent drinking (Neighbors et al., 2004). The results of our study are consistent with our previous finding of increased selection and consumption of vegetables following exposure to a descriptive social norm message in a laboratory setting (Robinson et al., 2014).

Here, it was also possible to study vegetable sales after removal of the poster and to observe a further increase in purchase of meals with vegetables. Given the pre-post design, an underlying time trend toward greater vegetable consumption, or a change in the type of customer toward those who are more inclined to choose vegetables cannot be ruled out. However, the stability in sales of water implies some consistency in purchasing behaviors over time, and the observational data suggest that the characteristics of the customer base did not differ significantly across time in terms of age, gender, and weight status (although these variables would benefit from being measured in more detail in future studies). The study was conducted across three separate workplace settings to reduce the overall variation in purchase patterns that might be introduced by examining a single site. There are several possible reasons why the increase in vegetable purchases was maintained after the posters were taken down. If a customer was prompted to purchase vegetables, and enjoyed eating those vegetables, then the behavior might have been reinforced, leading to a change in habit and/or a positive change in self-perception about vegetable liking and consumption. The social norm information might also have drawn attention to other people in the restaurant who were consuming vegetables, and there may have been some modeling of this behavior that was maintained after the intervention period (see Vartanian et al., 2015 for a review of modeling of eating behavior). Such an effect is predicted by the focus theory of normative conduct (Kallgren, Reno, & Cialdini, 2000), which suggests that normative information is most effective in guiding behavior when it is made salient or accessible. It is possible that observing other people consuming vegetables in the restaurant served as a cue to retrieve the normative information even after the posters were removed. A similar maintenance of the social norm effect was observed by Burger and Shelton (2011), who observed that people were more likely to use the stairs than the elevator for a week after intervention materials promoting stair use were taken down. However, to be sure that this latent effect is real, the findings would need to be replicated using a randomized controlled design.

Prior to conducting the present study, we were aware of only one other report of a significant effect of a social norm message in a field setting. Mollen and colleagues examined self-reported purchases, rather than sales, and an effect of the social norm message to increase vegetables purchases was found only for customers who reported noticing the posters (Mollen et al., 2013). Here, a positive association of a social norm message with cash register recorded purchases is reported, that holds for the entire sample. It is possible that a larger association would have been observed for those participants who noticed the specific message on the posters. There was no assessment of whether the selection of meals translated into consumption, but this seems likely given evidence that the majority of people in similar settings clear their plates (Hinton et al., 2013).

Very recently, Thorndike, Riis, and Levy (2016) conducted a randomized control trial to examine whether social norm feedback (a letter mailed to participants) with or without a financial incentive could increase the purchase of healthy food items in a hospital cafeteria. They reported that social norm feedback with an incentive, but not social norm feedback in isolation, produced a significant increase in healthy food choices. A possible reconciliation of the null effect reported by Thorndike and the significant results reported here is that, in line with the focus theory of normative conduct (Kallgren et al., 2000), social norms may have a greater effect on behavior when they are presented where the behavior takes place (e.g., a poster by a food counter where food is selected), but fail to exert a significant influence when they are not presented at this point (e.g., a letter sent to a participant). An important point to consider in the further development of social norms interventions aimed at increasing the purchase of vegetables is the extent to which the results may be transferable to a range of food outlets. Workplace restaurants in which customers are familiar with the other diners may lend themselves to social norm interventions because impression management concerns may motivate conformity to the norm (Herman et al., 2003). Hence, different types of restaurant may not yield the same results, and so further investigation is required to establish in which context social norm messages might be best targeted. One study placed placards on supermarket trolleys displaying the average number of produce items purchased at that supermarket and found that this increased purchase of produce items (Payne, Niculescu, Just, & Kelly, 2015). Both the present study and that conducted by Payne and colleagues used a norm message that emphasized the proximal context of the normative behavior (the other people in that location). Other evidence suggests that individuals are more likely to be influenced by descriptive norms that are derived from the setting those individuals are currently occupying (e.g., most people *here* choose to eat vegetables with their lunch; see Goldstein, Cialdini, & Griskevicius 2008). Establishing a connection between an individual and the norm referent based on their shared immediate surroundings might be sufficient to prompt following of the normative information.

The present results add to a growing body of data supporting the use of public health campaigns that have a basis in social norm theory (Marteau, Ogilvie, Roland, Suhrcke, & Kelly, 2001) and their advocacy (Davies et al., 2014). Evidence suggests that norm messaging maybe effective in reducing risky behaviors such as excessive alcohol intake and behavior harmful to the environment such as excessive energy consumption, although systematic reviews of the effectiveness of such campaigns have yielded inconsistent results and questions remain about when and for whom norm interventions may result in behavior change (see Miller & Prentice, 2016, for a review). Fewer studies have evaluated the effects of norm messages designed to enhance a health promoting behavior, although social norm interventions have been reported to enhance sunscreen use and increase levels of physical activity (e.g., Priebe & Spink, 2012; Reid & Aiken, 2013). An advantage of an intervention that emphasizes the positive, healthy behaviors of others is that resistance to such messages is less likely than for messages that use controlling language (e.g., you should eat vegetables because it is good for your health; Miller, Lane, Deatrick, Young, & Potts, 2007). Although the increased sale of meals with vegetables from the pre- to postintervention phase may appear small at 7%, it is similar to that reported for health communication campaigns (Ammerman, Lindquist, Lohr, & Hersey, 2002; Pomerleau, Lock, Knai, & McKee, 2005; Snyder, 2007), and if this approach were adopted more widely across workplace restaurants, it could impact a substantial number of meals. It is premature to comment on the clinical significance of our findings, especially given that we did not measure actual consumption and the long-term effects of the approach are unclear. However, evidence suggests that higher consumption of fruit and vegetables is associated with reduced risk of all-cause mortality, with an average reduction in risk of 5% for each additional vegetable serving a day (Wang et al., 2014). Overall, the results are promising because social norm interventions are likely to be cost-effective to implement (requiring only the resources to produce and print a message) and have the potential to reach consumers who might most benefit from increasing their consumption of vegetables, such as those consuming low levels of vegetables.

Future research should first establish, using randomized controlled designs, how the effect of the social norm message compares with a control intervention. In addition, food wastage associated with this intervention should be evaluated to confirm that the vegetables purchased with meals are actually consumed. If the effectiveness of the social norm message is confirmed, subsequent work might investigate ways of optimizing social norm interventions by testing the effects of different message types and possibly combining social norm interventions with other approaches to add value. Combining information about how others behave with information about whether that behavior is valued or endorsed by the social group may be particularly effective in prompting behavior change (Payne et al., 2015). Such an approach may also guard against the possibility that people who are already performing the behavior at the normative level will react by reducing positive behavior to fit in with the norm (Schultz, Nolan, Cialdini, Goldstein, & Griskevicius, 2007). It would be useful to examine whether any increases in vegetable purchase represented additions to the standard meal or a substitute for other components of the meal (e.g., reducing the amount of more energy dense foods purchased), since this will be critical to the net health impact. Future work should also distinguish between people who are habitual consumers of vegetables and those who are not habitual consumers of vegetables. At present we do not know if the norm message was associated with an increase in consumption of vegetables by those already consuming vegetables or by those who do not regularly consume vegetables. Previous data from laboratory-based studies suggest that low consumers might be more responsive to norm messages, but this remains to be tested in the field. Only a small proportion of the sample were observed in the present study and so it would be better in future studies to be able to provide demographic characteristics for the whole sample. In addition, a limitation of the study is that body weight was observed rather than measured, which may be subject to bias. Finally, it would also be desirable in future studies to examine a longer time period for the intervention, to know if the effectiveness of the posters diminishes over time due to customers habituating to their presence (although this might be offset by using different versions of the poster each week or changing their location). It would also be useful to assess a longer postintervention phase to examine whether behavior is maintained in the longer term.

The results of this study suggest that the social norm approach can be used to increase the purchase of vegetables and that further testing of its potential is warranted.

## Figures and Tables

**Table 1 tbl1:** Participant Characteristics and Meal Purchase Measures Split by Study Phase

Measure	Pre-intervention	Intervention	Post-intervention
Participant characteristics			
Male participants	54%	58%	60%
Not overweight	71%	76%	77%
Participants under 60 years of age	97%	99%	99%
Meal purchases			
Meals purchased with vegetables	1,897	2,028	2,070
Meals purchased without vegetables	1,281	1,151	1,018
Total meals purchased	3,178	3,179	3,088
Percentage of meals purchased with vegetables	60%	64%	67%
Cold drink purchases			
Purchases of water	744	738	740
Purchases of other cold drinks	4,895	5,292	5,228
Total cold drinks purchased	5,639	6,030	5,968
Percentage of water purchased	13%	12%	12%
